# HEALTH CARE ACCESS AND USE AMONG ADULTS AGING WITH INTELLECTUAL AND DEVELOPMENTAL DISABILITIES

**DOI:** 10.1093/geroni/igad104.1848

**Published:** 2023-12-21

**Authors:** Jeffrey E Stokes, Danielle A Waldron, Jacquelin Sauer, Emily Hartford

**Affiliations:** University of Massachusetts Boston, Boston, Massachusetts, United States; Stonehill College, North Easton, Massachusetts, United States; Stonehill College, Easton, Massachusetts, United States; Stonehill College, Easton, Massachusetts, United States

## Abstract

Adults with intellectual and developmental disabilities (IDD) are living longer than ever before, a testament to advancements in healthcare. However, adults with IDD face a number of challenges accessing healthcare, and particularly preventative healthcare targeted towards midlife and older adults, which have previously been far less utilized by adults aging with IDD. This study uses longitudinal data from the National Core Indicators In-Person Survey (NCI-IPS) to examine factors associated with adults with IDD’s utilization of two preventative healthcare measures specific to mid- and later life: mammograms and colonoscopies. Both mammogram and colonoscopy utilization exhibited curvilinear age trajectories, with usage increasing through ages 60 and 65 respectively, before decline at older ages. For both mammogram and colonoscopy utilization, living with family was associated with a 50% lower likelihood of utilization, while poor health, access to transportation, and community inclusion were associated with increased usage. For mammograms, participating in a community activity was also associated with increased utilization. Women with IDD were also about 25% less likely to receive a colonoscopy than men. Interestingly, the likelihood of receiving a mammogram dropped significantly in 2018-2019 compared to a decade prior, whereas the likelihood of receiving a colonoscopy increased over time. No racial/ethnic differences were found. Results highlight a number of factors associated with preventative healthcare use among the population of adults aging with IDD. These were similar for mammogram and colonoscopy utilization, and highlight the importance of living situation, transportation, and community engagement for adults with IDD’s access to preventive medicine.

